# Supplementary material for: COMPASS subunit Bre2 regulates chromatin remodeler Arp9 to control Aspergillus flavus aflatoxin synthesis and virulence
Source: Nat Commun. 2026 Feb 20;17:1862. doi: 10.1038/s41467-026-69877-0 (PMC12923686; doi:10.1038/s41467-026-69877-0)
Supplement: Supplementary file 1 — Supplementary Information [file 41467_2026_69877_MOESM1_ESM.pdf]

COMPASS subunit Bre2 regulates chromatin remodeler Arp9 to control *Aspergillus flavus* aflatoxin synthesis and virulence

Zhuang *et al.*

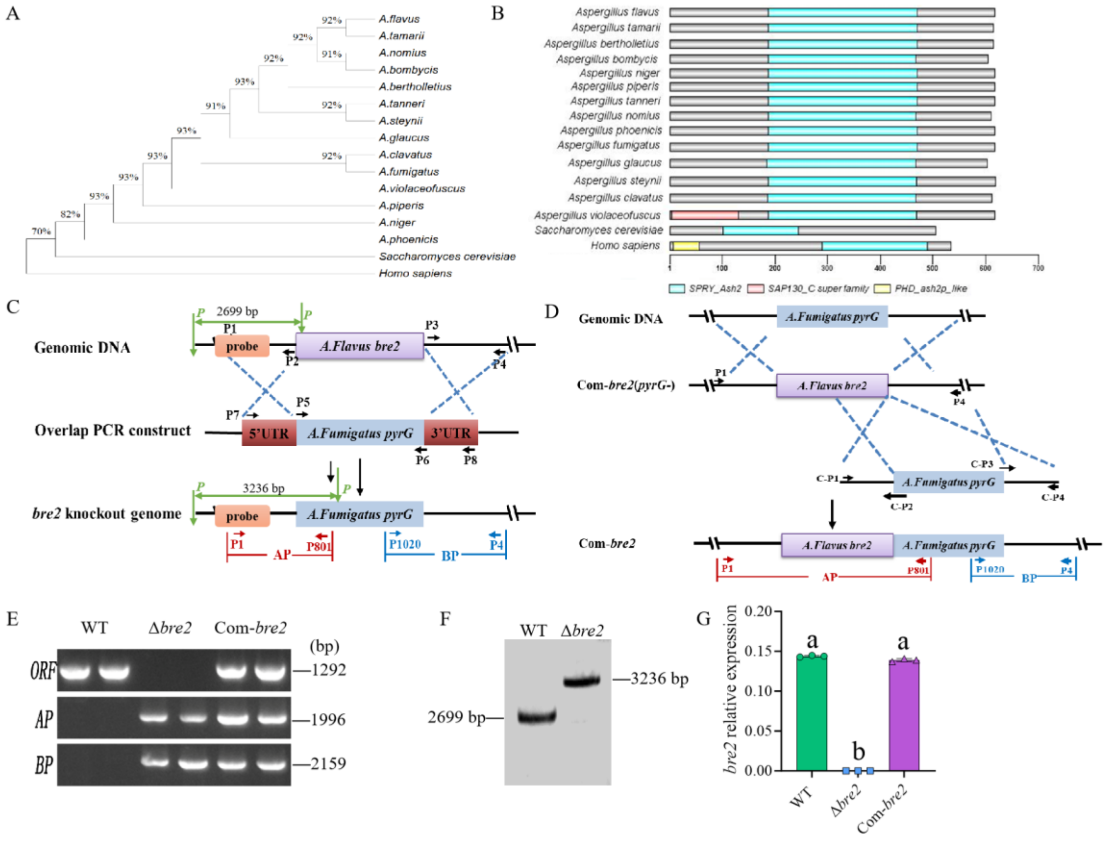

Supplementary Figure 1. Bioinformatics analysis of Bre2 and the construction of *bre2* deletion and its complementary strains. (A) Evolutionary analysis of Bre2. The Bre2 orthologs of model species, including *A. flavus*, *A. tamarii*, *A. nomius*, *A. bombycis*, *A. bertholletius*, *A. tanneri*, *A. steynii*, *A. glaucus*, *A. clavatus*, *A. fumigatus*, *A. violaceofuscus*, *A. piperis*, *A niger*, *A phoenicis*, *Saccharomyces cerevisiae* and *Homo sapiens* were aligned, and the evolutionary tree was drawn by MEGA11.0. (B) The analysis on the domains of Bre2 orthologs by software SMART, and the identified domains were visualized using IBS 1.0. The domain of SPRY_Ash2, SAP130_C super family and PHD_ash2p_like were indicated in blue, red and yellow, respectively. (C-D) The strategy for the construction of *bre2* deletion and complementary strains. In the preparation of *bre2* deletion and complementary strain, the 5'FR-*bre2*-3'FR fragment was amplified with primers P1 and P4. In the transformation process, 5-FOA (5-fluoroorotic acid) was added to inhibit the strain containing *pyrG*. (E) The constructed Δ*bre2* and Com-*bre2* strains were validated by PCR with genomic DNA as template. DNA fragment AP was amplified with primer p1 and p801, BP with primer p1020 and p4, and ORF with primer p9 and p10, as shown in the strategy panel (C and D). (F) The *bre2* gene mutant (Δ*bre2*) was identified by Southern blotting analysis. Genomic DNA from WT and Δ*bre2* strains were digested by *Pst1* (presented by *P* in the construction scheme of the C panel) and hybridized with a 1.35 kb probe (the 5'-flanking region of *bre2*), and the probe was amplified with primers probe-F and probe-R. (G) The expression level of *bre2* in the above fungal strains at 37°C was monitored with RT-qPCR. Data in (G) are presented as mean ± SD (n = 3). One-way ANOVA coupled with Tukey's multiple comparisons test was used in statistical significance analysis for panel G. The little letters above the columns show significant differences (P<0.05). Source data are provided as a Source Data file.

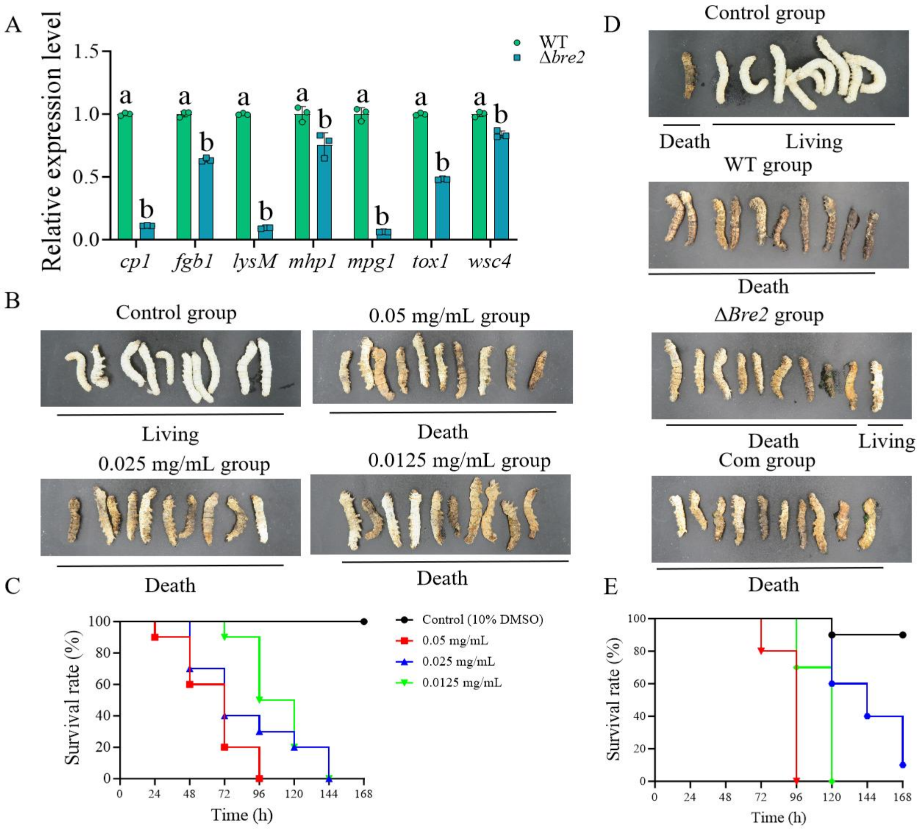

Supplementary Figure 2. Analysis of key fungal virulence factors regulated by Bre2. (A) Relative expression levels of virulence and colonization associated effectors of fungi. (B) The silkworms were daily injected with 5 μL AFB1 standard (dissolved in 10% DMSO) at different concentrations (0.05 mg/mL, 0.025 mg/mL and 0.0125 mg/mL) until the 7th day. (C) The survival rate of the above silkworms in one-week post-injection with aforementioned AFB1 solution (n = 10). (D) The silkworms were injected with 10% DMSO dissolved AFs extracted from Δ*bre2,* Com-*bre2* and WT strain each day 5 μL. (E) The survival rate of the above silkworms after injection with aforementioned AFs was monitored in 1 week (n = 10). Data in (A) are presented as mean ± SD (n = 3). The unpaired two-tailed t-test was used to compare the statistical significance of panel A. Statistical analysis between groups in panel E was using the log-rank test. The little letters above the columns show significant differences (P<0.05), and *** means significant difference of P<0.001. Source data are provided as a Source Data file.

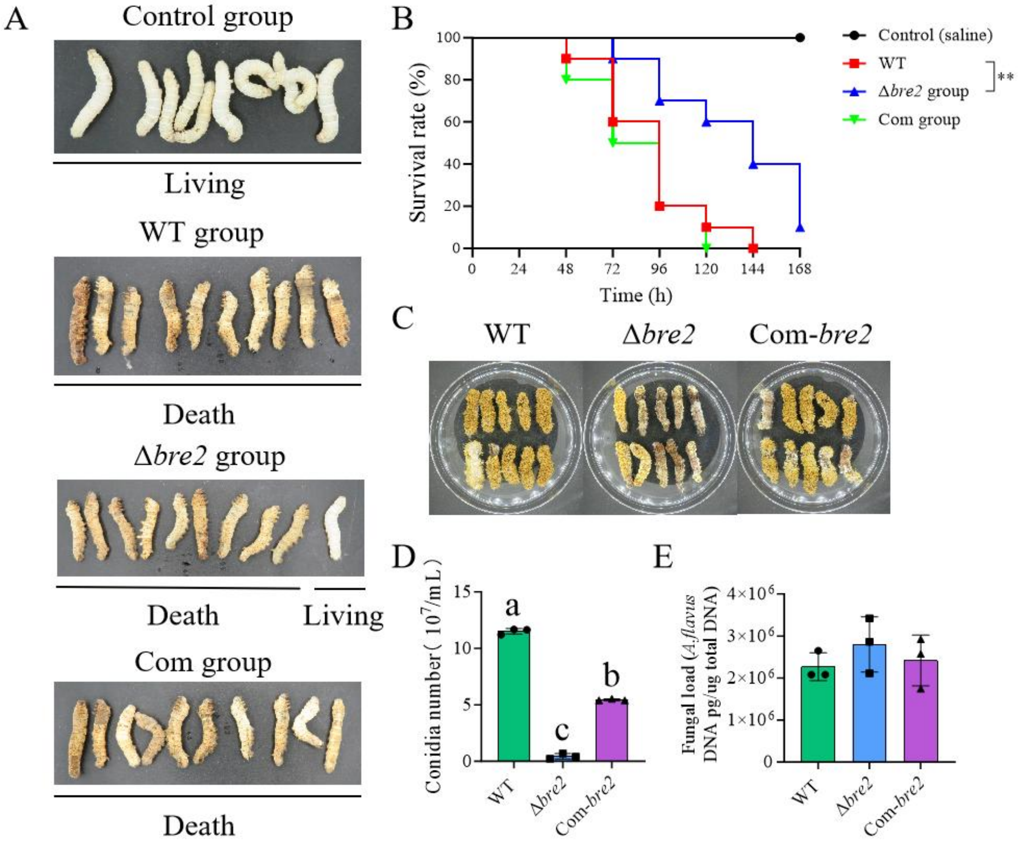

Supplementary Figure 3. Assessment of the fungal virulence to silkworm mediated by Bre2. (A) Photographs of the silkworms infected with 5 μL (10^7spores/mL) of the spores of WT, Δbre2 and Com-bre2 strains after 1 week incubation. (B) The survival rate of silkworms after 1 week injection of the above strains (n = 10). (C) The dead silkworms infected by the above strains after 7 d incubation in dark under 29°C. (D) The histogram showing the conidia number on the dead silkworms according to panel C. (E) The mycelial biomass in dead silkworms was assessed by quantification of the fungal DNA with qRT-PCR. Data in (D and E) are presented as mean ± SD (n = 3). Statistical analysis between groups in panel B were using the log-rank test. One-way ANOVA coupled with Tukey's multiple comparisons test was used in statistical significance analysis for panel D and E. The little letters above the columns show significant differences (P<0.05), and ** means significant difference of P<0.01. Source data are provided as a Source Data file.

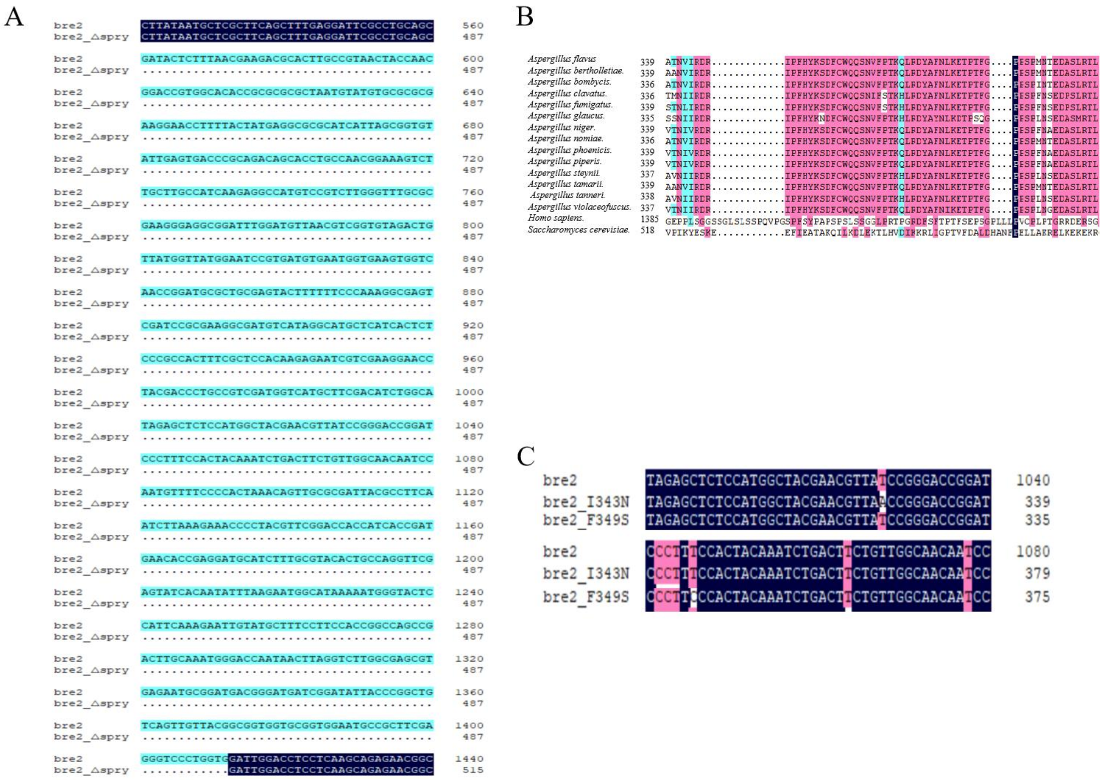

Supplementary Figure 4. Construction of SPRY domain knockout strain and its conserved amino acid residue mutant strains. (A) Sequencing and sequence alignment of the SPRY knockout strain with *A. flavus Bre2*. (B) Multiple sequence alignment results of the amino acid sequence of Bre2 orthologs from all species. (C) The results of sequence alignment of *A. flavus bre2* with the mutant strains *bre2*^{I343N} and *bre2*^{F349S}.

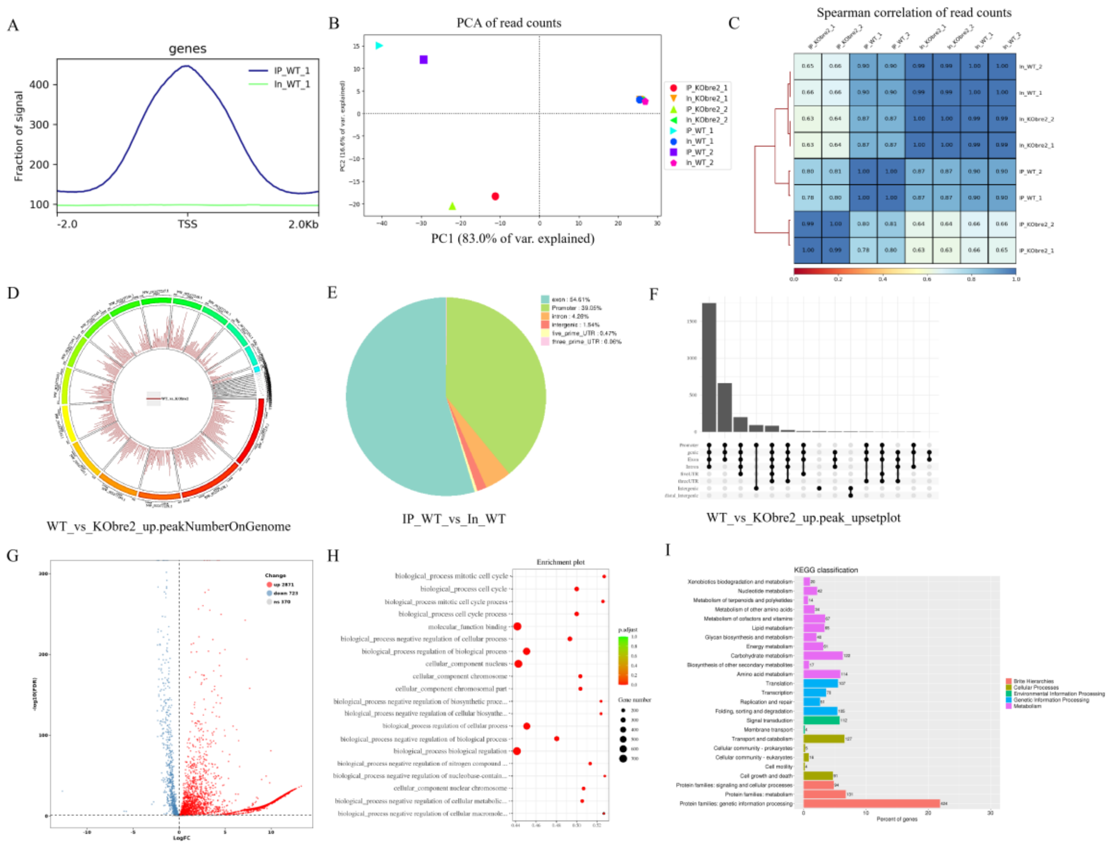

Supplementary Figure 5. ChIP-seq analysis on the H3K4me3 modified chromatin fragments catalyzed by Bre2. (A) Density distribution of reads in the 2 kb region upstream and downstream of the TSS of the gene in the WT strain. (B) PCA analysis among samples in the ChIP-seq analysis. (C) Correlation analysis among the ChIP-seq samples. (D) The distribution of DAPs on the *A. flavus* genome. (E) Distribution of peaks of H3K4me3 modified chromatins according to the functional elements of the genome genes of *A. flavus*. (F) The functional elements joint analysis on the distribution of DAPs on *A. flavus* genome. (G) Distributions of $\log_2$ FC (WT vs Δ*bre2*), and 2871 DAPs for all 3964 identified peaks are significantly accumulated in the WT samples compared to the Δ*bre2* samples (Red color; $-\log_2$ FC ≥ 1, $P<0.01$), 723 DAPs are down-regulated in the WT samples compared to the Δ*bre2* samples (blue color; $\log_2$ FC$\leq$-1, $P<0.01$), and 370 peaks are not differentially accumulated (gray color; $\log_2$ FC<1 or >-1, $P>0.01$ if $\log_2$ FC>1 or <-1). (H) The annotation of biological processes, cellular components and molecular function for the genes which are up-modified at the WT strain compared to the Δ*bre2* strain. (I) The annotation of KEGG pathways of up-modified genes in the WT strain compared to the Δ*bre2* strain.

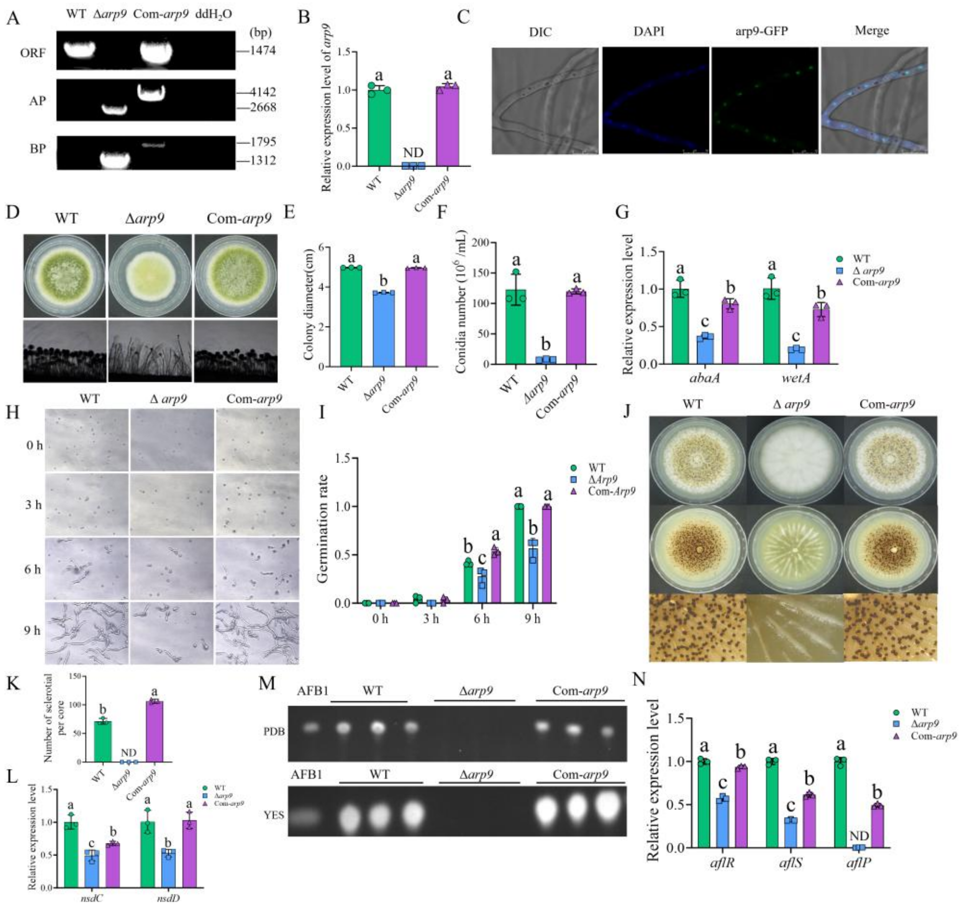

Supplementary Figure 6. Construction and phenotypes of *arp9* mutant strains. (A) The constructed fungal mutants, including the Δ*Arp9* and Com-*Arp9* strains were identified with the diagnostic PCR analysis. (B) The expression level of *arp9* in the above fungal strains at 37°C was monitored with qRT-PCR. (C) Subcellular localization of Arp9 in *A. flavus* mycelium under laser confocal microscopy. (D) The colonies of the WT, Δ*Arp9* and Com-*Arp9* strains grown on PDA at 37°C in dark for 5 d. (E) The statistics of colony diameter for the above fungal strains. (F) The statistics of spore production for the above fungal strains. (G) The transcriptional expression levels of *abaA* and *wetA* genes in the WT, Δ*Arp9* and Com-*Arp9* fungal strains at 48 h. (H) Spore germination for the WT, Δ*Arp9* and Com-*Arp9* strains in PDA medium at 0 h, 3 h, 6 h and 9 h. (I) Statistics analysis on spore germination rates of the above strains. (J) The WT, Δ*Arp9* and Com-*Arp9* strains were point-inoculated on CM medium at 37°C in dark for 7 d (the above panels), then, were sprayed with 75% ethanol (the middle panels), and the middle panels were enlarged under a dissecting microscope (the lower panels). (K) Statistics of sclerotium number for the WT, Δ*Arp9* and Com-*Arp9* strains. (L) The transcriptional expression levels of sclerotium-formation related genes *nsdC* and *nsdD* in the WT, Δ*Arp9* and Com-*Arp9* strains. (M) The biosynthesis of AFB1 in the WT, Δ*Arp9* and Com-*Arp9* strains were detected by TLC after being grown on PDB and YES at 29°C in dark for 7 d. (N) Transcriptional levels of aflatoxin-related genes *aflR*, *aflS* and *aflP* from the WT, Δ*Arp9* and Com-*Arp9* strains. Data in (B, E-G, I, K, L, and N) are presented as mean ± SD (*n* = 3). One-way ANOVA coupled with Tukey's multiple comparisons test was used in statistical significance analysis for panel B, E-G, I, K, L and N. The little letters above the columns show significant differences (*P*<0.05), and ND means not detected. Source data are provided as a Source Data file.

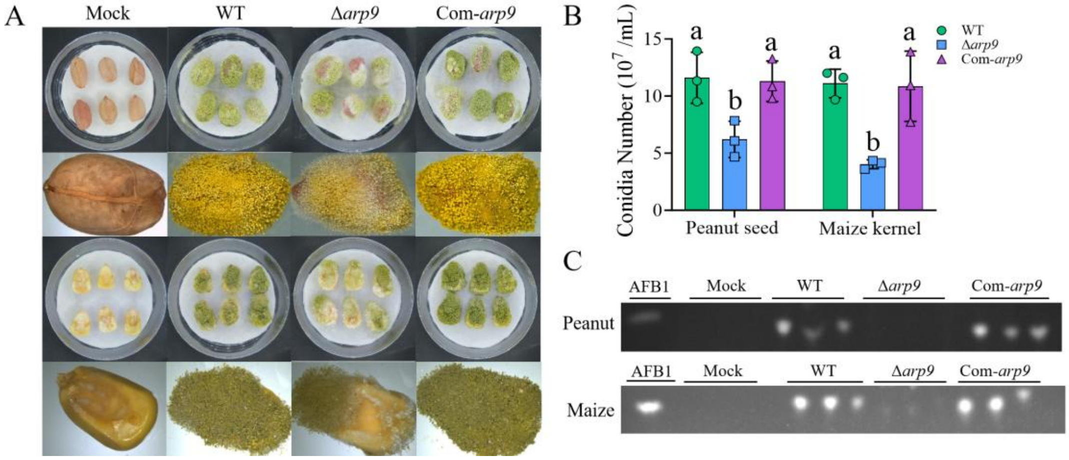

Supplementary Figure 7. Function of Arp9 in colonization of host crops by *A. flavus*. (A) Phenotypic analysis of peanut seeds and corn kernels colonized by *A. flavus* WT, Δ*Arp9*, and Com-*Arp9* strains at 29℃ in dark for 7 d. (B) Statistical analysis of the conidia number of *A. flavus* on the surface of peanut and maize kernels. (C) TLC analysis of AFB1 yield in peanut seeds and maize kernels infected by the above fungal strains after 7 d inoculation. Data in (B) are presented as mean ± SD (n = 3). One-way ANOVA coupled with Tukey's multiple comparisons test was used in statistical significance analysis for panel B. The little letters above the columns show significant differences (P<0.05). Source data are provided as a Source Data file.

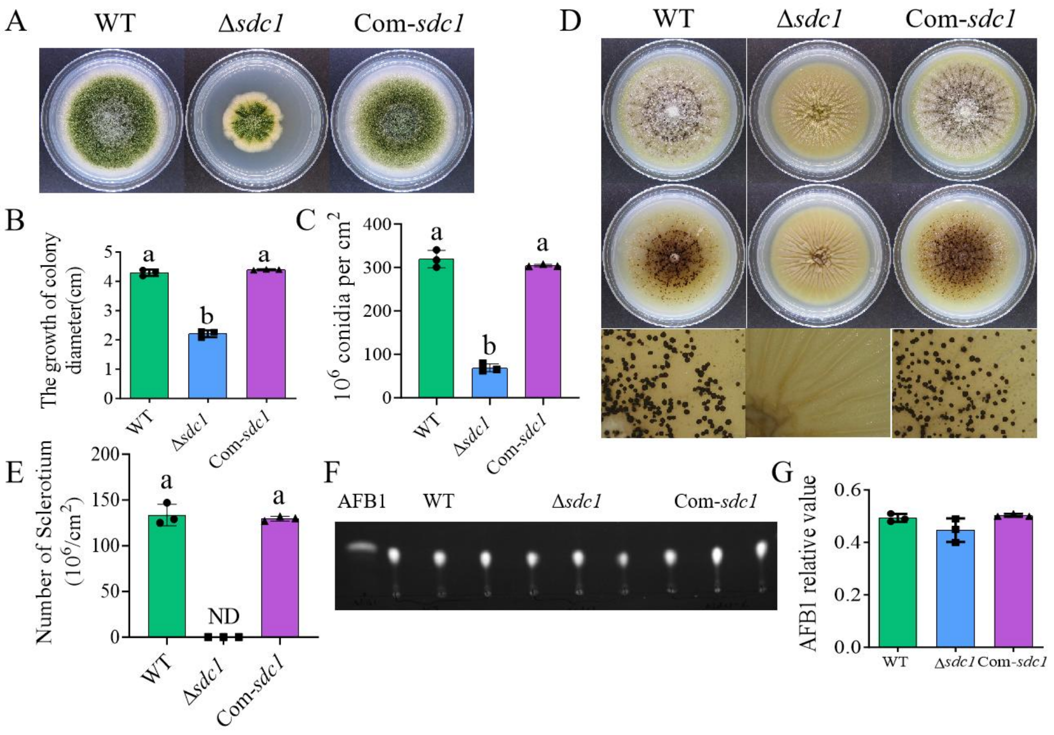

Supplementary Figure 8. Sdc1 participates in the regulation of fungal morphogenesis but is not involved in AFB1 biosynthesis. (A) The colonies of the WT, Δ*sdc1* and Com-*sdc1* strains grown on PDA at 37°C in dark for 5 d. (B) The statistics of colony diameter for the above fungal strains. (C) The statistics of spore production for the above fungal strains. (D) The WT, Δ*sdc1* and Com-*sdc1* strains were point-inoculated on CM medium at 37°C in dark for 7 d (above panels). Middle panels were sprayed with 75% ethanol, and the lower panels were the enlarged pictures of the middle panels under a dissecting microscope. (E) Statistics of sclerotia number for the WT, Δ*sdc1* and Com-*sdc1* strains. (F) The biosynthesis of aflatoxins in the WT, Δ*sdc1* and Com-*sdc1* strains were detected by TLC after being grown in PDB at 29°C in dark for 7 d. (G) Relative aflatoxin production in the TLC analysis was quantified. Data in (B, C, E, and G) are presented as mean ± SD (n = 3). One-way ANOVA coupled with Tukey's multiple comparisons test was used in statistical significance analysis for panel B, C, E and G. The little letters above the columns show significant differences (P<0.05), and ND means not detected. Source data are provided as a Source Data file.

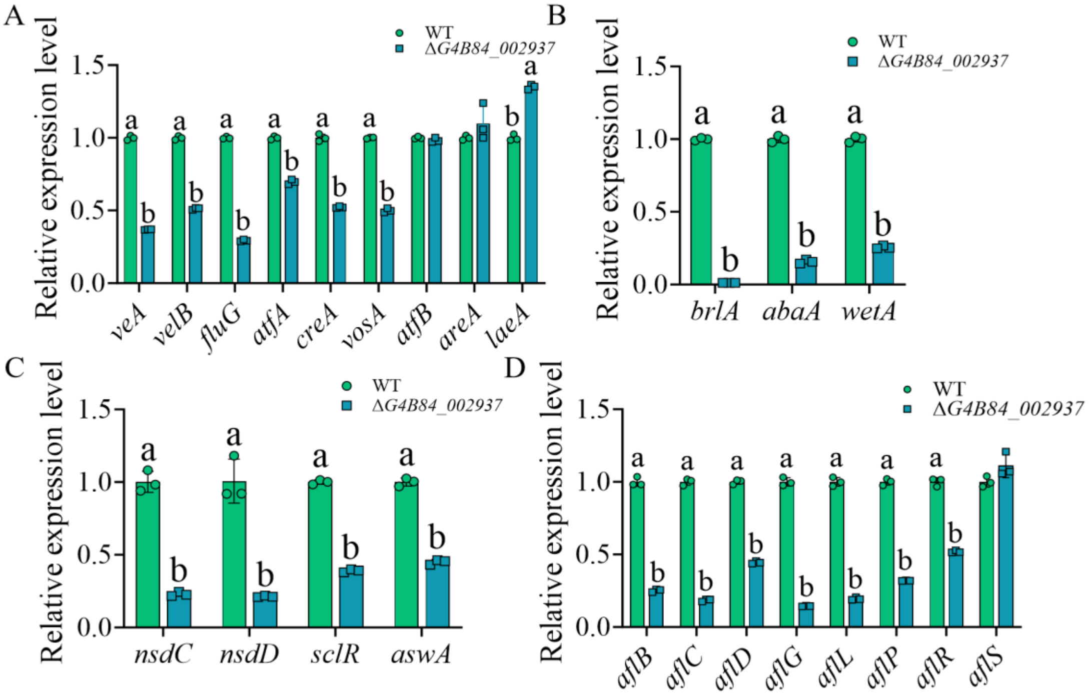

Supplementary Figure 9. Exploration of SMAP (G4B84_002937) regulation pathway. (A) Relative expression levels of global regulators (including *veA*, *velB*, *fluG*, *atfA*, *creA*, *vosA*, *atfB*, *areA*, and *laeA*) in the WT and ΔG4B84_002937 strains. (B) The transcriptional expression levels of sporulation-related genes (*brlA*, *abaA* and *wetA*) in the WT and ΔG4B84_002937 strains. (C) The transcriptional expression levels of sclerotia-formation related genes (*nsdC, nsdD, sclR* and *aswA*) in the WT and ΔG4B84_002937 strains. (D) Transcriptional levels of aflatoxin-related genes (*aflB*, *aflC*, *aflD*, *aflG*, *aflL*, *aflP*, *aflR* and *aflS*) in the WT and ΔG4B84_002937 strains. Data in (A-D) are presented as mean ± SD (n = 3). The unpaired two-tailed t-test was used to compare the statistical significance. The little letters above the columns show significant differences (P<0.05). Source data are provided as a Source Data file.
