# Supplementary material for: COMPASS subunit Bre2 regulates chromatin remodeler Arp9 to control Aspergillus flavus aflatoxin synthesis and virulence
Source: Nat Commun. 2026 Feb 20;17:1862. doi: 10.1038/s41467-026-69877-0 (PMC12923686; doi:10.1038/s41467-026-69877-0)
Supplement: Supplementary file 3 — Description of Additional Supplementary Files [file 41467_2026_69877_MOESM3_ESM.pdf]

Description of Additional Supplementary Files

File Name: Supplementary Data 1
Description: The total of 63 proteins were pulled down by Arp9.

File Name: Supplementary Data 2
Description: The strains used in this study.

File Name: Supplementary Data 3
Description: The primers used in fungal strain construction and verification.

File Name: Supplementary Data 4
Description: The primers for qRT-PCR in this study.

File Name: Supplementary Data 5
Description: The primers for CHART-qPCR in this study.
